# General Medicine and Therapeutics

**Published:** 1893-05

**Authors:** 


### GENERAL MEDICINE AND THERAPEUTICS.

EDITED BY DR. LUTHER B. GRANDY.

### THERAPEUTIC MERIT OF COMBINED REMEDIES.

In nearly every case where quinia is indicated it can be advantageously combined with antikamnia, which thus becomes a valuable adjunct to quinia. Quinia, for example, is a most decided febrifuge, and its action is usually promptly reliable; but when combined with this member of the aromatic series its action is markedly increased. Some individuals, however, cannot take any of the coal-tar derivatives; consequently the use of antikamnia will be inhibited in such cases; on the other hand, some patients cannot take quinine.

An important benefit to be derived from the addition of antikamnia to quinine is that it removes the sense of fullness in the head, constriction about the forehead and tinnitus aurium—so common when the latter drug is administered alone; the disturbing action of quinia on the auditory nerve is suspended to a great extent, and the usual quinine deafness is absent. The combination of these agents in tablet form is a happy one.

The combination of antikamnia with quinia is valuable in the racking headache, with high fever, attendant upon malarial disorders. It is likewise valuable in cases of periodical attacks of headache of non-defined origin; of the so-called "bilious attacks"; of dengue; in neuralgia of the trigemini; in that of "ovarian catarrh"; and, in short, in nearly every case where quinine would ordinarily be prescribed.

Binz claims specific antiseptic powers for quinia; other writers are in accord with him on this point, and report good results from large doses in septicæmia, pyæmia, puerperal fever and erysipelas. It is a germ destroyer of the bacilli of influenza (la grippe). A full dose of quinine and antikamnia will promptly relieve many cases of this disease. In the gastric catarrh of drunkards this combination is valuable. Quinia is a poison to the minute organism—sarcina; and antikamnia exerts a soothing, quieting effect on the nerve filaments. A full dose of antikamnia and quinia will often arrest a commencing pneumonia or pleuritis. This combination is also useful in the typho-malarial fever of the South—particularly for the hyperpyrexia—both quinia and antikamnia, as previously said, being decided fever reducers.

The germicide power of quinia is the explanation of its success in the treatment of malarial disturbances. Thus it is also a prophylatic against the various manifestations of malarial poison, and, as such, can be relied on. The cause of malaria as a disease consists of pigmented bodies, which penetrate the interior of the red blood corpuscles—pigmented bodies of various shapes and flagellate organisms—both having amœboid movements—the filaments being in active vibration.

In miningeal troubles, attended by marked acceleration of the heart due to the rise in the fever temperature, full doses of quinine and antikamnia at intervals of, say, about four hours, will be productive of good. In measles, large doses of the combination at night—say ten grains of each for adults (dose for children in proportion), will relieve the distress of the catarrhal pneumonia, and modify, in a great degree, the amount of the exudative products. The periodical neuroses which may be either regular or irregular in their manifestations, but which are dependent on the malarial germ for their origin, are all controllable by the combination of quinine and antikamnia. Examples of such neuroses are asthma, laryngismus stridulus, summer catarrh, etc. Indeed, for the hemicrania and neuralgias of malarial origin the combination of quinine and antikamnia, just alluded to, may be declared *a specific.*

The dose of quinine may be made smaller than usual when administered with antikamnia. Thus one or two tablets of two and a half grains each of quinine and antikamnia will prove sufficient for great utility in puerperal mania, in the headaches of advanced age, accompanied with vertigo and despondency.

This combination is capable, by the combined influence of each drug on the nervous system and blood, of restraining all the processes which develop heat, organic changes and muscular motion; therefore it is "the one thing needful" in the treatment of the hyperpyrexia of malarial fevers. In the vast majority of cases, when necessary to administer quinine, if antikamnia be added to the prescription the results will be surprising.

Formerly the idea prevailed that in order to render the treatment of periodical fevers efficient the gastro-intestinal tube should be cleaned out by emetics and cathartics. This, however, is a fallacy, as the conditions they are intended to remove depend mainly on the malarial poison, for which the combination of quinine and antikamnia is the specific cure.

In speaking of the treatment of pneumonia by quinine and antikamnia Prof. Palmer says: "The effects desired, and certainly as a rule produced, are a decided reduction of temperature, a marked diminution in the frequency of the pulse, a decided moisture of the skin or free sweating, a slower and more easy respiration, or relief from pain and the feeling of fullness of the chest, a diminution of the cough and of the tenacious and bloody character of the expectoration; and, in short, not only is there a checking of the fever, but of all evidences—general and local—of the pulmonary engorgement and inflammation."

In Meniere's disease, or "labyrinthine vertigo," this combination has, by persistent use, entirely removed the trouble in many cases. The curative effects of quinine and the coal-tar antipyretics in sunstroke are well known, and have been used recently with great benefit in numerous instances in this country and in India. In hysteria, and even in epilepsy, the combination of quinine and antikamnia is often indicated, and will frequently give the desired results. In whooping cough and hay fever quinine and antikamnia will prove beneficial.

The tablets of equal parts of quinine and antikamnia spoken of in this article can be administered by the rectum with good effect. They should first be dissolved in whisky, and then water can be added in any quantity needed—always remembering the total quantity of each drug in such enemata.—*Dr. Stephen J. Clark in Virginia Medical Monthly.*

---

### SECTION OF GENERAL MEDICINE OF THE PAN-AMERICAN MEDICAL CONGRESS.

This unique assemblage promises to be one of the most important events that has occurred in the history of medicine in the Americas. Its success is assured by the large number of valuable papers already promised. The Section on General Medicine, which is one of the most important that has been created, bids fair to be one of the most successful in the entire Congress; and already many valuable contributions are in process of preparation, and will be read at the meeting in September. It is hoped, with the hearty co-operation of all physicians living not only in North but also in South and Central America, that the work in this section will be memorable; and each physician living on this continent is requested to join this most important section, and to prepare a contribution to be read before that body. It is especially requested that those intending to join this section or to read papers shall at once send their names, with titles of papers, to the Secretary, Dr. Judson Daland, No. 319 South Eighteenth Street, Philadelphia, Pa., so that they may be noted on the calendar and given their appropriate places.

---

Dr. J. B. S. Holmes, of Rome, will not be deterred by the fire which totally destroyed his sanitarium some time ago. He is vigorously at work on new buildings which will probably be ready for use by November 1st. In the meantime he has leased some residenses in which he is now prosecuting his work. We hope that his loss will ultimately be a gain.

